# The Expression Quantitative Trait Loci in Immune Response Genes Impact the Characteristics and Survival of Colorectal Cancer

**DOI:** 10.3390/diagnostics12020315

**Published:** 2022-01-26

**Authors:** Ren-Hao Chan, Po-Chuan Chen, Yu-Min Yeh, Bo-Wen Lin, Kai-Di Yang, Meng-Ru Shen, Peng-Chan Lin

**Affiliations:** 1 Department of Surgery, National Cheng Kung University Hospital, College of Medicine, National Cheng Kung University, Tainan 704, Taiwan; n803421@mail.hosp.ncku.edu.tw (R.-H.C.); cpc324@gmail.com (P.-C.C.); linbw@mail.ncku.edu.tw (B.-W.L.); 2 Department of Oncology, National Cheng Kung University Hospital, College of Medicine, National Cheng Kung University, Tainan 704, Taiwan; s98031083@mail.ncku.edu.tw; 3 Department of Computer Science and Information Engineering, College of Electrical Engineering and Computer Science, National Cheng Kung University, Tainan 704, Taiwan; n150956@mail.hosp.ncku.edu.tw; 4 Department of Obstetrics and Gynecology, National Cheng Kung University Hospital, College of Medicine, National Cheng Kung University, Tainan 704, Taiwan; 5 Graduate Institute of Clinical Medicine, National Cheng Kung University Hospital, College of Medicine, National Cheng Kung University, Tainan 704, Taiwan; 6 Department of Pharmacology, National Cheng Kung University Hospital, College of Medicine, National Cheng Kung University, Tainan 704, Taiwan; 7 Department of Genomic Medicine, National Cheng Kung University Hospital, College of Medicine, National Cheng Kung University, Tainan 704, Taiwan

**Keywords:** expression quantitative trait loci, tumor microenvironnement, immune response genes, *FCER1G*, colorectal cancer

## Abstract

The impact of germline variants on the regulation of the expression of tumor microenvironment (TME)-based immune response genes remains unclear. Expression quantitative trait loci (eQTL) provide insight into the effect of downstream target genes (eGenes) regulated by germline-associated variants (eVariants). Through eQTL analyses, we illustrated the relationships between germline eVariants, TME-based immune response eGenes, and clinical outcomes. In this study, both RNA sequencing data from primary tumor and germline whole-genome sequencing data were collected from patients with stage III colorectal cancer (CRC). Ninety-nine high-risk subjects were subjected to immune response gene expression analyses. Seventy-seven subjects remained for further analysis after quality control, of which twenty-two patients (28.5%) experienced tumor recurrence. We found that 65 eQTL, including 60 germline eVariants and 22 TME-based eGenes, impacted the survival of cancer patients. For the recurrence prediction model, 41 differentially expressed genes (DEGs) achieved the best area under the receiver operating characteristic curve of 0.93. In total, 19 survival-associated eGenes were identified among the DEGs. Most of these genes were related to the regulation of lymphocytes and cytokines. A high expression of *HGF*, *CCR5*, *IL18*, *FCER1G*, *TDO2*, *IFITM2*, and *LAPTM5* was significantly associated with a poor prognosis. In addition, the *FCER1G* eGene was associated with tumor invasion, tumor nodal stage, and tumor site. The eVariants that regulate the TME-based expression of *FCER1G*, including rs2118867 and rs12124509, were determined to influence survival and chromatin binding preferences. We also demonstrated that *FCER1G* and co-expressed genes in TME were related to the aggregation of leukocytes via pathway analysis. By analyzing the eQTL from the cancer genome using germline variants and TME-based RNA sequencing, we identified the eQTL in immune response genes that impact colorectal cancer characteristics and survival.

## 1. Introduction

Many researchers have shown their interest in the impact of germline variants on clinical outcomes in different types of cancer. The common variants typically have modest effects on entire biological pathways. In contrast, some rare variants are believed to lead to dysregulation in biological systems [1]. The identification of prognostic and predictive germline variants for cancer research is a developing field. For example, the germline *SUFU* variant is believed to predispose an individual to medulloblastoma and is associated with a poor prognosis [2]. One hallmark of cancer is that some cancer cells evade the host’s immune system. The immune system is an essential part of the tumor microenvironment (TME). The immune response genes that regulate germline variants play a crucial role in the clinical outcomes of cancer patients [3]. For example, the expression of PD-L1/PD-1 in the TME contributes to tumor suppression and enhances the immune tolerance of tumor cells. By understanding the role of immune cells in the TME, we can develop new immunotherapies for currently nonresponsive tumors.

Transcription has substantial genetic control. Expression quantitative trait loci (eQTL) provide insight into the effect of downstream target genes (eGenes) regulated by trait-associated variants (eVariants). They provide a molecular basis for the phenotype–genotype association. In a cancer study, a comprehensive eQTL analysis revealed the target genes in cancer susceptibility loci from genome-wide association studies (GWASs) [4,5]. Germline variants caused by somatically acquired or inherited gene alterations can lead to immune response gene expression in the TME [6]. From the eQTL analysis of TME-based immune gene expression, we identified survival-associated eQTL and determined how immune cells influence cancer risk, development, and prognosis.

By applying technologies such as immune response gene RNA sequencing (RNA-seq) with germline whole-genome sequencing and patients’ clinical information, we attempted to reveal the relationships between eVariants in TME-based immune response eGenes and clinical outcomes through eQTL analyses. This study explores not just the function of gene expression but also the mechanisms of gene regulation. Importantly, the underlying germline eVariants could mold the TME-based immune response eGenes expression to affect the cancer characteristics and survival. eVariants were potential biomarkers for the prediction of cancer recurrence. eGenes were implied as potential therapeutic targets.

## 2. Materials and Methods

### 2.1. Enrollment of Cancer Patients

This was a cohort study. Eligible cancer patients were aged ≥20 years and had histologically confirmed pathological stage II–III colorectal cancer (CRC), an Eastern Cooperative Oncology Group performance status (ECOG PS) of 0–1, and adequate organ function. Patients were willing to provide blood and cancer tissue samples for research purposes. The exclusion criteria were as follows: patients receiving chemotherapy within six months, with other malignancies, and with a life expectancy of less than one year. A total of 124 patients with colorectal cancer (CRC) were recruited for the study at the National Cheng Kung University Hospital (NCKUH) between January 2015 and January 2019. Follow-up continued through August 2020. Clinical information and blood and tissue samples for DNA extraction were collected at the time of enrollment. The NCKUH Institutional Review Board approved this study (A-ER-103-395, A-ER-104-153, and B-ER-109-154), and all participants provided written informed consent.

### 2.2. Whole Blood Cell Whole-Genome Sequencing

Genomic DNA from collected blood samples was quantified with a Qubit fluorescence assay (Thermo Fisher Scientific, Waltham, MA, USA) and sheared with an S2 instrument (Covaris, Woburn, MA, USA). Library preparation was conducted using the TruSeq DNA PCR-Free HT Kit (Illumina, San Diego, CA, USA). Individual DNA libraries were measured using the 2100 Bioanalyzer (Agilent, Santa Clara, CA, USA) qPCR and Qubit (Thermo Fisher Scientific). All flow cells were sequenced on a HiSeq 2500 sequencer (Illumina) using the SBS kit V4 chemistry (Illumina). FastQC was used for quality control, and the resulting reads were aligned to the hg19 reference genome with the BWA-MEM algorithm. The identification of SNPs and indels and genotyping were performed across all samples simultaneously using standard hard-filtering parameters or variant quality score recalibration according to the GATK Best Practices recommendations. WGS was performed using a minimum median coverage of 30×.

### 2.3. RNA-Seq Library Preparation, Quantification, Pooling, and Sequencing

Cancer tissues with immune response gene expression profile data were obtained from 99 CRC patients. RNA was prepared from formalin-fixed paraffin-embedded (FFPE) tissue extracted with the RecoverAll Total Nucleic Acid Isolation Kit (Thermo Fisher Scientific). RNA concentration was determined on an Invitrogen™ Qubit™ Fluorometer with the Qubit™ RNA High Sensitivity Assay (Thermo Fisher Scientific). Twenty nanograms of RNA was used for each reverse transcription reaction, and cDNA was prepared with the SuperScript™ IV VILO™ Master Mix Kit. Immune response libraries were prepared using the Ion AmpliSeq™ Kit for Chef DL8 with the Ion Chef™ System and the instructions in the Oncomine™ Immune Response Research Assay user guide (Pub. No. MAN0015867). The raw gene expression data were preprocessed using Torrent Suite (Thermo Fisher Scientific), followed by further normalization. Twenty-two samples failed quality control because of insufficient sequenced reads.

### 2.4. WGS and RNA-Seq Data Preprocessing

Briefly, genotyped variants were excluded based on the following criteria: (i) Hardy–Weinberg equilibrium *p*-value < 1 × 10^−6^ estimated from the Hardy–Weinberg R package [7], (ii) minor allele frequency < 0.05, and (iii) location on a non-autosomal chromosome or chromosome Y. Immune response genes were selected on the basis of expression reads ≥6 in at least 20% of samples. Expression reads were normalized using the size factors calculated using the DESeq2 R package [8] followed by quantile normalization.

### 2.5. Differentially Expressed Gene Analysis

Differentially expressed genes were identified using the DESeq2 R package [8] with *p*-values < 0.05.

### 2.6. Comparison of Gene Expression between Different Types of Samples

Normalized RNA-seq data from the TCGA and GTEx project were downloaded from a cohort comprised of TCGA, GTEx, and TARGET provided by the UCSC-Xena platform (http://xena.ucsc.edu/ (accessed on 13 April 2020)) [9]. The Wilcoxon rank-sum test was used to compare different types of samples.

### 2.7. Dichotomization for Gene Expression

An optimal cutoff point for each gene’s dichotomizing expression level was identified with the function survcutpoint built-in the survminer R package [10].

### 2.8. cis-eQTL Mapping

We mapped cis-eQTL for all preprocessed variants within ±1 megabase of each gene’s transcriptional start site (TSS) using an additive linear model built-in Matrix eQTL [11]. We included only sex and age as covariates because of the simple ethnic composition. Significant eQTL were defined with the threshold of a raw *p*-value < 1 × 10^−3^.

### 2.9. In Comparison with the GTEx Database

The loci of our result were first transformed to the reference genome hg38 via the LiftOver tool built in the UCSC Genome Browser [12] to compare with Single-Tissue cis-QTL Data V8 downloaded from the GTEx portal (https://gtexportal.org/home/ (accessed on 21 May 2020)). The overlapping cis-eQTL were identified if the eVariants, eGenes, and signs of the slope were the same as those from the GTEx.

### 2.10. Statistical Analysis

Fisher’s exact test and odds ratios were used to assess the relationship between dichotomized gene expression and clinical features, including the tumor invasion stage, tumor nodal stage, location, and tumor mutational burden. Kaplan–Meier curves were used to evaluate recurrence-free survival combined with *p*-values derived from the log-rank test. The Cox proportional hazards model built-in survival R package [13] was used to assess the hazard ratio of each potential immune response gene. Recurrence-free survival was defined as the time between surgery and cancer recurrence.

### 2.11. Machine Learning

The caret R package [14] was used to build all machine learning models, including the SVM, logistic regression, random forest, and multilayer perceptron. To estimate each model’s performance, the AUROC, accuracy, sensitivity, specificity, negative predictive value, positive predictive value, and F1 score were derived from the 3-fold cross-validation repeated 100 times.

### 2.12. Linkage Disequilibrium

Linkage disequilibrium (LD) was estimated by the genetics R package [15], and the LD map was generated using the LD heatmap R package [16].

## 3. Results

### 3.1. Patient Characteristics and Study Design

**Figure 1:**
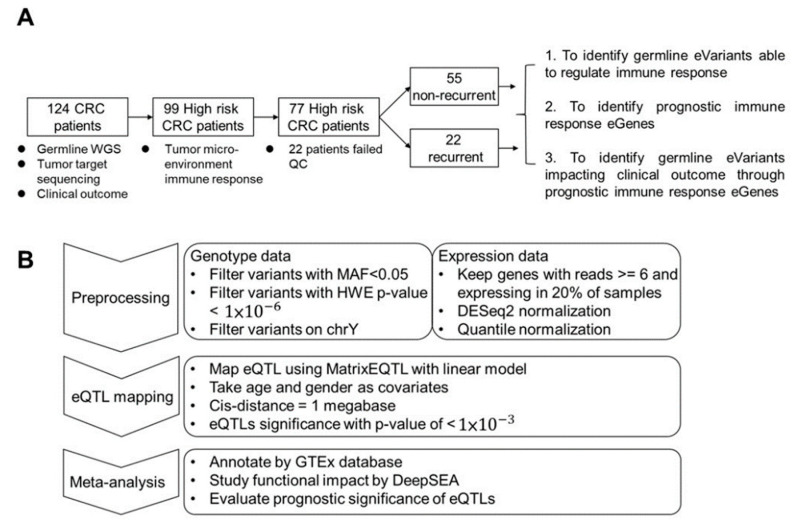
Study design and workflow for cis-eQTL mapping. (**A**) Study design. Overall, 126 CRC patients were enrolled in this study. Ninety-nine patients were subjected to RNA-seq analysis. After filtering low-quality data, 77 patients were kept for further study with three aims. The first was to identify cis-eQTL in patients’ tumor micro-environment. In the second, prognostic immune response genes were identified and used to cluster patients. Finally, eVariants that impacted patient survival through immune response eGenes were identified for further investigation. (**B**) Workflow of eQTL mapping. For the first step, variants derived from WGS with Hardy–Weinberg equilibrium *p*-values < 1 × 10^−6^, a minor allele frequency among these patients of <0.05, or a location on non-autosomal chromosome or chromosome Y were excluded. Next, expression data were normalized via DESeq2 and quantile normalization. Next, the R package Matrix eQTL was used to map eQTL using age and sex as covariates, and the cis distance was set to 1 megabase (Mb). For the meta-analysis, known eQTL in the GTEx database, the functional impact predicted by DeepSEA, and prognostic significance were annotated to the resulting eVariants.

In this study, we enrolled 124 patients with stage III CRC. From all subjects, we collected germline whole blood cells for whole-genome sequencing (WGS) and primary tumor tissue for deep targeted sequencing. To study the impact of TME-based immune response-associated gene expression on recurrence, tumor samples were collected from 99 high-risk subjects. After quality control, 77 subjects remained for further analysis, among whom 22 patients (28.5%) suffered from tumor recurrence. We hypothesize that response eQTL impact the clinical outcome through the TME response. First, we identified germline eVariants that regulate immune response eGene expression by eQTL mapping analyses [11]. Second, we identified prognostic immune response eGenes. Third, we demonstrated that germline eVariants affect the clinical outcome through immune response eGenes (Figure 1A).

The baseline characteristics of the patients are reported in Table S1. A total of 50.6% of the patients were male. The median age of these patients was 58 years. The most common primary tumor location was the left colon (76.6%). The tumor invasive stage of these patients was high (T stage: T3–T4) (87%), while the tumor nodal stage was low (N stage: N0–N1) (70%). Thirty-six percent and ninety percent of patients had somatic nonsynonymous mutations in *KRAS* and *TP53*, respectively. The tumor mutational burden (TMB) was also estimated, and the top 10th percentile cutoff of TMB was considered a hypermutated status [17]. The distribution of age, sex, tumor location, tumor invasion stage, tumor nodal stage, *KRAS* mutations, *TP53* mutations, and hypermutation status were not significantly different between patients with/without tumor recurrence.

### 3.2. Mapping the Germline eVariants That Regulate Immune Response eGenes Expression

**Figure 2:**
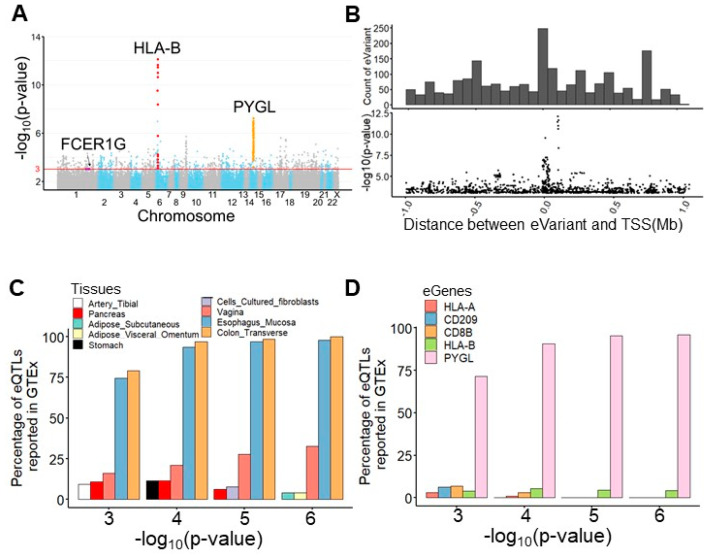

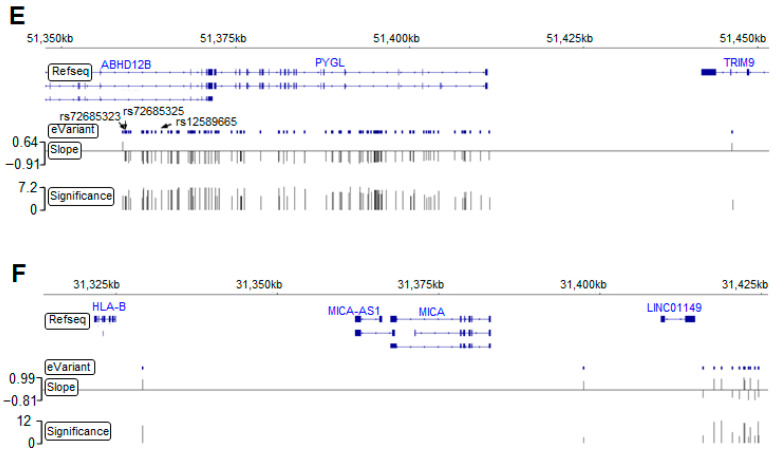
Germline eVariants that regulate immune response eGene expression by eQTL mapping analyses. (**A**) Manhattan plot of all cis-eQTL. The red line indicates significance (with a *p*-value of 1 × 10^−3^). Red, orange, and purple circles indicate the eQTL related to *HLA-B*, *PYGL*, and *FCER1G*, respectively. (**B**) Histogram showing the distribution of eQTL distances in megabases (Mb) from the transcription start site (TSS). Scatter plot showing the distribution of eQTL significance and distance from the TSS. (**C**) Histogram showing tissues sharing the same eQTL in the GTEx. With the significance increasing, overlapping eQTL were most frequently found in the esophageal mucosa and transverse colon. (**D**) Histogram showing overlapping eGenes in the GTEx. *PYGL* was the most frequently observed eGene in the GTEx, with a high significance. (**E**) Case study for *PYGL*. The eVariants track shows the eVariants that regulate the expression of *PYGL* (chr14:51371934–51411428) through our pipeline. The sloping track indicates the slope derived from eQTL mapping. A positive slope indicates that gene expression was upregulated through the gain of alternative alleles. The significance track indicates the negative log-transformed *p*-values derived from eQTL mapping. Predictive eVariants reported by Bien et al. (rs72685323, rs72685325, and rs12589665) were associated with the downregulation of *PYGL*, with slopes of −0.697 (*p* = 4 × 10^−5^), −0.675 (*p* = 4.36 × 10^−5^), and −0.847 (*p* = 6.05 × 10^−7^), respectively. (**F**) Case study for *HLA-B*. The eVariants track shows the eVariants that regulate the expression of *HLA-B* through our pipeline.

For expression quantile trait loci mapping, we performed an eQTL mapping analysis of TME-based immune response gene RNA-seq data and whole-genome sequencing data from 77 subjects using Matrix eQTL [18] (Figure 1B). After preprocessing, 7,596,484 germline variants and the expression levels of 378 immune response transcripts were retained for eQTL mapping. To realize the clinical and biological significance of the resulting eQTL, the public eQTL database, survival significance, and functional impact were integrated into our result. The log-rank test was applied to assess the survival significance of the resulting eVariants. The functional impact of these eVariants was evaluated by predicting their chromatin effects using DeepSEA, a deep learning-based algorithmic framework [19]. Overall, 94,220 cis-eQTL comprised 88,847 eVariants, and 377 eGenes with a *p*-value of <0.05 were identified (Table S2). These eVariants were spread throughout the intergenic (46.1%), intronic (41.5%), ncRNA (6.8%), up/downstream (2.7%), UTR (1.7%), and exonic (1.1%) regions of the genome. In addition, 51.8% of exonic eVariants were nonsynonymous variants. After setting a *p*-value < 1 × 10^−3^ as a threshold, 2083 significant eQTL, composed of 2063 eVariants and 241 eGenes, were retained for further analyses. Among these eQTL, the most significant eQTL were associated with eGene *PYGL*, the glycogen phosphorylase evolved in glycogen metabolism induced by hypoxia in solid tumors [20], and HLA-B, one of the human leukocyte antigen class I molecules whose expression is associated with tumor stage and the survival of CRC [21] (Figure 2A,B). Considering the clinical significance, 65 eQTL comprising 60 eVariants with *p*-values of <0.05 from the log-rank test and 22 eGenes were determined. Moreover, 146 eVariants with high probability (>0.7) of an SNP being an eQTL variant were identified by DeepSEA (Table S3).

### 3.3. PYGL and HLA-B eGenes Were Identified by the Genotype-Tissue Expression Project (GTEx)

We next explored the Genotype-Tissue Expression project (GTEx) database [22] and found 130 (6.2%) overlapping eQTL pairs with the same eVariants, eGenes, and regulatory trends in 2083 significant eQTL (Figure S1A). Intriguingly, our result and the GTEx database shared more overlapping eQTL in the transverse colon and esophageal mucosa compared with other tissues (Figure 2C). In addition, we found that the most significant overlapping eQTL were related to the *PYGL* and *HLA-B* eGenes (Figure 2D). To assess the roles of *PYGL* and *HLA-B* in CRC, we further examined the expression of these two genes sequenced from normal and tumor samples. RNA-seq data from The Cancer Genome Atlas (TCGA) and the Genotype-Tissue Expression project (GTEx) revealed that both *PYGL* and *HLA-B* were expressed at higher levels in normal tissues adjacent to colon cancer than in tumor tissues or normal tissues derived from subjects without cancer (Figure S1B). This result implies that *PYGL* and *HLA-B* are activated in the TME. Recently, Bien et al. showed that *PYGL* was significantly associated with CRC. They also identified rs12589665 and its spanning region covering two variants, rs72685325 and rs72685323, as a predictive locus for CRC [23]. Although these variants were identified in our study (Figure 2E), the high allele frequency (approximately 0.4) indicates that they are common in East Asian-related ancestry and might not be suitable for use as cancer-susceptible risk factors. We also identified eVariants rs2266161, rs10947210, rs62395278, rs2516455, and rs3093971, which were associated with the *HLA-B* eGene (Figure 2F) and have been reported in the GTEx database. However, there was no evidence about whether they were cancer-susceptible risk factors. Despite the importance of *PYGL* and *HLA-B*, as shown in previous studies, these two genes were not found to be prognostic factors in the patients described herein. We further conducted several analyses to identify potential prognostic molecules for clinical usage.

### 3.4. Identifying Essential Immune Response eGenes by Tumor Characteristics and Prognosis

#### 3.4.1. Differentially Expressed Genes (DEGs) Were Identified by Cancer Recurrence

**Figure 3:**
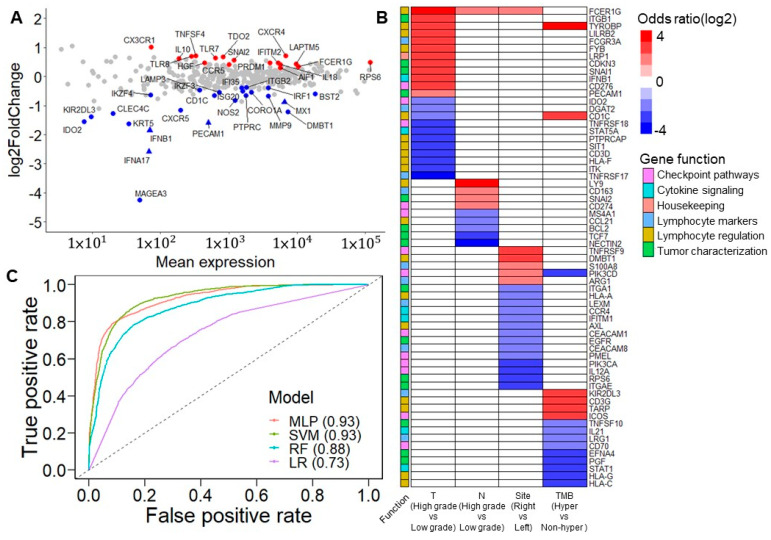
Prognostic immune response eGenes and tumor characteristics. (**A**) M (log ratio)-A (mean average) plot for the differentially expressed genes (DEGs) between patients with/without recurrence. Red points indicate the upregulated DEGs in patients with recurrence. Blue points indicate downregulated DEGs in patients with recurrence. Triangles indicate the significant DEGs after FDR correction. Most of the downregulated DEGs, including *IFNB1*, *MX1*, *ISG20*, *CXCR5*, *BST2*, *IFI35*, and *IRF1*, are involved in interferon signaling. However, lymphocyte regulators such as *CX3CR1*, *CXCR4*, *TLR8*, *TLR7*, *CCR5*, *IL18*, *FCER1G*, and *LAPTM5* are slightly upregulated in patients with recurrence. (**B**) Association between dichotomized immune response genes and clinical features. The association was derived from Fisher’s exact test. The color scale indicates the odds ratio. Red indicates that a high expression positively impacts tumor invasion, tumor nodal stage, tumor originating from the right side of the colon, and hypermutation status. Blue indicates that high expression negatively impacts these clinical features. White is used when the association is not significant. (**C**) Area under the receiver operating characteristic curve (AUROC) of five classification models. Classification models were built from 41 DEGs. Among these strategies, the multilayer perceptron and support vector machine models had the best performance, with an AUROC of 0.93 (MLP: multilayer perceptron; SVM: support vector machine; RF: random forest; LR: logistic regression).

A differential gene expression analysis was performed using the DESeq2 R package [8] to observe the difference in the immune response in the TME in patients with and without tumor recurrence. As a result, 41 differentially expressed genes (DEGs) were identified (Figure 3A, Table S4). Based on the categorization of the Oncomine immune response research assay, the results showed that most of the downregulated immune response genes, including *IFNB1*, *MX1*, *ISG20*, *CXCR5*, *BST2*, *IFI35*, and *IRF1*, were involved in the signaling of interferons, which have multiple immunoregulatory effects [24]. It has been demonstrated that the absence of interferons leads to the metastasis of tumor cells [25]. Alternatively, lymphocyte regulators such as *CX3CR1*, *CXCR4*, *TLR8*, *TLR7*, *CCR5*, *IL18*, *FCER1G*, and *LAPTM5* were slightly upregulated in patients with recurrence.

#### 3.4.2. Correlation of 41 DEGs and Tumor Characteristics

The expression levels of all genes were divided into high and low groups using maximally selected rank statistics [26] to evaluate each gene’s clinical relevance and prognostic significance (Table S5). After adjusting the *p*-values of the log-rank test that compared the dichotomized expression of each gene by Benjamini–Hochberg adjustment, 36 were found to be significant, with a false discovery rate of <0.05 (Figure S2). These data showed that tumor invasion stage, tumor nodal stage, tumor location, and TMB were influenced by particular genes (Figure 3B). Among these results, a high expression of *FCER1G* was related to the high T and N stages—namely, the proliferation and migration of tumor cells. Additionally, a high *FCER1G* expression was observed more in the right side of the colon than in the left side of the colon. In addition, a previous study demonstrated a similar association of CD274 and CD3G considering lymph node metastases [27] and hypermutation [28], respectively.

#### 3.4.3. Predictive Ability of 41 DEGs for Cancer Recurrence in Machine Learning Model

In terms of features importance, we applied several machine learning strategies, including the support vector machine (SVM), random forest, logistic regression, and multilayer perceptron (MLP), to analyze the dichotomized expression of 41 DEGs and estimate their performance in predicting the cancer recurrence state. On comparing patients with and without recurrence, the SVM and MLP achieved the best performance, with an area under the receiver operating characteristic curve (AUROC) of 0.93 (Figure 3C). Regarding sensitivity, the SVM and MLP performed better than the other strategies, with scores of 0.78 and 0.77, respectively. Except for logistic regression, all the different models reached a specificity score of more than 0.9 (Figure S3A). The results confirm that 41 DEGs are essential features in molecular biology predicting cancer recurrence.

#### 3.4.4. 36 Survival-Associated Genes Were Selected in Machine Learning Model

Alternatively, we applied the same machine learning strategies to analyze the dichotomized expression of the 36 most significant survival-associated genes. The performance of the MLP, SVM, and random forest was similar, with AUROCs of approximately 0.88, better than logistic regression with an AUROC of 0.64 (Figure S4A). Both the SVM and MLP had the best sensitivity of 0.68, which was lower than that of the models that incorporated 41 DEGs. The random forest showed the highest specificity of 0.92, followed by the MLP (Figure S3B). By applying K-means fuzzy clustering, these patients were grouped into the protective group. Only one patient experienced relapse, and the risk group contained most patients with recurrence (Figure S4B). Compared with the protective group, it was observed that some lymphocyte regulatory factors were upregulated and some cytokine signaling factors were downregulated in the risk group. The results indicate the usefulness of combining cancer recurrence and prognostic survival factors to identify potential prognostic-associated eQTLs (eVariants and eGenes).

### 3.5. Germline eVariants Affect the Clinical Outcome through Immune Response eGenes

#### 3.5.1. Selecting Survival-Associated eVariants and eGenes form 41 DEGs

Integrally speaking, 19 survival-associated eGenes among the DEGs were subjected to a detailed examination (Table S6). Most of these genes were related to the regulation of lymphocytes and cytokines. The results indicated that a high expression of *HGF*, *CCR5*, *IL18*, *FCER1G*, *TDO2*, *IFITM2*, and *LAPTM5* was significantly associated with a poor prognosis (Table 1). Previous studies also revealed that most of these genes substantially impacted the prognosis of patients with various types of cancer and were potential targets for clinical usage [29,30,31]. Combined with previous eQTL analyses, we found nine eQTLs, including nine eVariants and two eGenes (*HGF* and *FCER1G*), that correlated with the survival of cancer patients. Among these potential target genes, the expression of *FCER1G* was related to clinical relevance, such as the tumor invasion stage, tumor nodal stage, and primary tumor location. Therefore, we used *FCER1G* as the candidate eGene for further validation.

#### 3.5.2. Validation of eVariants and FCER1G eGene by TCGA and GTEx

**Figure 4:**
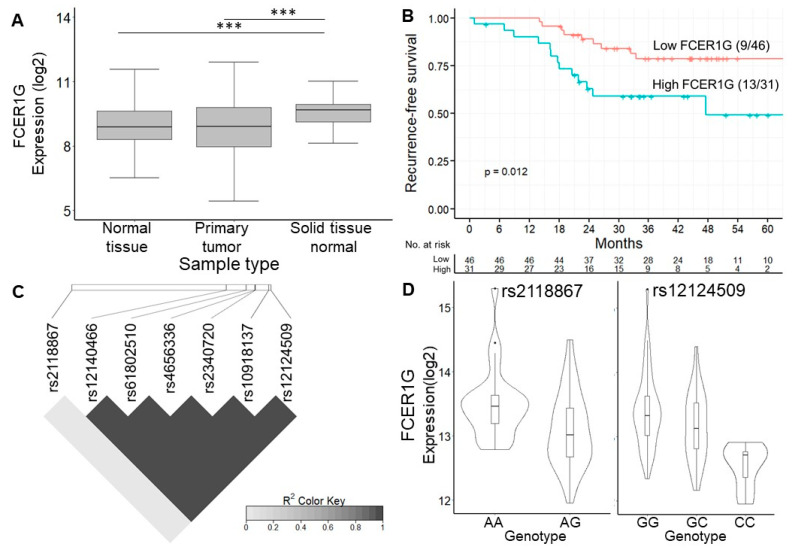
Expression of FCER1G eGene and its eVariants. (**A**) Box plot showing the expression level of *FCER1G* between different studies. The expression of *FCER1G* was higher in cancer tissues than in normal tissues. Our results showed that *FCER1G* was highly expressed in the primary tumors of these CRC patients. (**B**) Kaplan–Meier curve showing the difference in recurrence-free survival between patients with high or low *FCER1G* expression. High expression of *FCER1G* was related to a poor prognosis, with a log-rank *p* = 0.012. (**C**) Linkage disequilibrium map of 7 *FCER1G*-related eVariants. The functionally significant eVariant rs12124509 was in linkage disequilibrium with the other eVariants (r^2^ > 0.99), except for the survival-associated eVariant rs2118867 (r^2^ < 0.1). (**D**) Violin plot showing the expression level of *FCER1G* according to different genotypes of rs2118867 and rs12124509. The downregulation of *FCER1G* was related to the gain of alternative alleles of rs2118867 and rs12124509 and the other eVariants in LD with the former. *** *p*-value < 1 × 10^−3^ from Wilcoxon rank sum test.

*FCER1G* is believed to be involved in cancer prognosis. Bulk RNA-seq data from the TCGA and GTEx project showed that the expression of *FCER1G* was higher in normal tissues near colon cancer than in tumor tissue or normal tissue from noncancer subjects (Figure 4A). In addition, the analysis of the Human Protein Atlas [32] revealed not only that the *FCER1G* protein was expressed on the cytoplasmic membrane of colon cancer cells by immunohistochemistry staining but also that a high expression level of *FCER1G* was associated with poor survival, which is consistent with our results (Figure 4B, a log-rank *p* = 0.012). In order to control the effects of other clinical features such as age and gender, we conducted multivariate cox regression to investigate the significance of FCER1G eGenes. The results indicated the increased recurrent risk of high FCER1G expression compared to low FCER1G expression in the study cohort (adjust HR: 3.4; 95% CI 1.26–9.37). The details of multivariate cox regression were shown in Table S7. The number of patients at risk was appended to the Kaplan–Meier plot in Figure 4B. These results imply that the high expression of *FCER1G* in the tumor microenvironment (TME) is a prognostic factor. The eVariant rs12124509 was predicted to influence the binding of chromatin, with the smallest functional significance score from DeepSEA. In contrast, the log-rank test revealed that rs2118867 was a survival-associated eVariant, with a *p*-value of <0.05 (hazard ratio = 0.429). In addition, we surprisingly found that rs12124509 was in perfect linkage disequilibrium (LD) with the other eVariants (r^2^ > 0.99), except for rs2118867 (r^2^ < 0.1) (Figure 4C). The allele frequency of rs1214509 and variants in LD are higher in non-Finnish European, Finnish, and Ashkenazi Jewish populations (0.648 to 0.715) than in African and East Asian populations (0.142 to 0.304). These data indicate that alternative alleles do not frequently occur in Africans and East Asians compared to other populations. Alternatively, the worldwide allele frequency of rs211867 is similar in different populations ranging from 0.426 (African) to 0.578 (Ashkenazi Jewish). However, the allele frequency of rs211867 is 0.318 in the population of Taiwan, which is lower than that of other populations. The downregulation of *FCER1G* was associated with the gain of alternative alleles of the survival-associated eVariant rs2118867, and the most functional eVariant rs12124509 and the other eVariant in LD with the former (Figure 4D). These results indicate that alternative alleles of rs211867, rs12124509, and their spanning LD regions lead to a good prognosis by decreasing *FCER1G* expression in the TME.

### 3.6. The Biological Process of FCER1G and Its Co-Expressed Immune Genes

**Figure 5:**
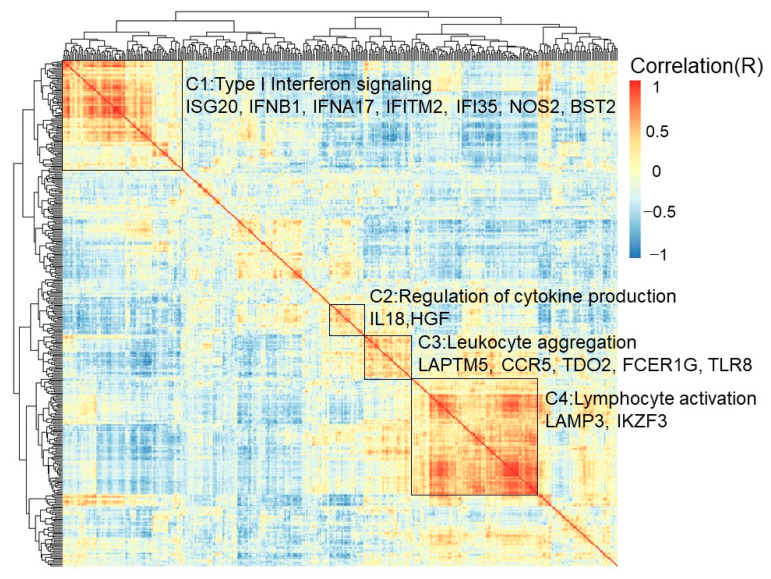
Biological process of FCER1G and its co-expressed immune genes. Clustered heatmap showing the co-expressed immune response genes and related biological processes. *FCER1G* and its co-expressed immune response genes were grouped into cluster 3, which implied leukocyte aggregation. Type I interferon signaling was enriched with genes in cluster 1, including the prognostic *ISG20*, *IFNB1*, *IFNA17*, *IFITM2*, *IFI35*, *NOS2*, and *BST2* eGenes. Cytokine production regulation was enriched with genes in cluster 2, including the prognostic *IL18* and *HGF* eGenes. Leukocyte aggregation genes, including the prognostic *LAPTM5*, *CCR5*, *TDO2*, *FCER1G*, and *TLR8* eGenes, were enriched in cluster 3. Lymphocyte activation genes, including the prognostic *LAMP3* and *IKZF3* eGenes, were enriched in cluster 4.

The univariate odds ratio model was used to study immune gene expression levels and revealed the high expression of *FCER1G.* The overexpression of the top five genes—*SRGN*, *HAVCR2*, *AIF1*, *TNFSF4*, and *GZMA*—was correlated with a high *FCER1G* expression. The downregulation of the top five genes—*STAT6*, *GADD45GIP1*, *LEXM*, *BCL2*, and *ICOSLG*—was associated with a high *FCER1G* expression (Figure S5). Pathway analysis was performed using DAVID [33]. There are four major signaling pathway clusters: cluster 1 to cluster 4 (Figure 5). Type I interferon signaling genes, including *ISG20*, *IFNB1*, *IFNA17*, *IFITM2*, *IFI35*, *NOS2*, and *BST2* eGenes, were enriched in cluster 1. A higher expression of *IFITM2* may be regulated by rs764825643. *IFITM2* is associated with lymphatic metastasis and poor clinical outcomes [34]. Cytokine production regulation genes, including the *IL18* and *HGF* eGenes, were enriched in cluster 2. A higher expression of *HGF* may be regulated by rs6962550/rs11365736. *HGF* could activate ERK1/2 and AKT via MET phosphorylation, resulting in cetuximab resistance in colorectal cancer patients [35]. Leukocyte aggregation genes, including the prognostic *LAPTM5*, *CCR5*, *TDO2*, *FCER1G*, and *TLR8* eGenes, were enriched in cluster 3. Chemokine receptor 5 (*CCR5*) is associated with liver metastasis [36]. The results indicate that targeting the *CCR5* axis can be an effective strategy. *TLR8* expression increased the chemotherapy resistance in colorectal cancer. *FCER1G* and its co-expressed immune response genes were related to leukocyte aggregation and poor survival (Figure 5). Lymphocyte activation genes, including the *LAMP3* and *IKZF3* eGenes, were enriched in cluster 4. A high *LAMP3* protein expression was significantly associated with the migration and invasion of tumor cells in vitro, lymph node metastasis, and poor overall survival [37].

## 4. Discussion

We conducted a comprehensive analysis of eQTL from the cancer genome using germline variants and TME-based RNA sequencing, potentially identifying the eQTL with *FCER1G* in immune response genes that impact colorectal cancer characteristics and survival. Germline genetic variants are associated with an increased risk of cancer development. However, the genetic variants that affect the clinical outcome through TME-based immune response gene expression are still unknown in CRC. We integrated whole-genome sequencing data and TME-based immune response gene expression data through eQTL mapping to address these issues. These analyses also provided potential eQTL, including germline genetic variants and immune gene expression, to predict the prognosis and determine possible biological mechanisms. Our results highlight the following important points: (i) 65 eQTL, including 60 germline eVariants in 22 TME-based eGenes, impact the clinical outcome; (ii) 49 TME-based immune response gene panels can be used to predict prognosis; (iii) 19 survival-associated eGenes were identified among the DEGs (most of these genes are related to the regulation of lymphocytes and cytokines); (iv) 9 eQTL, including 9 eVariants and 2 eGenes (*HGF* and *FCER1G*), correlated with the survival of cancer patients; (v) eQTL, including the rs2118867 and rs12124509 eVariants in TME-based *FCER1G* eGene expression, influence both colorectal cancer clinical characteristics and survival; and (vi) *FCER1G* and its co-expressed immune response genes are related to leukocyte aggregation genes such as *LAPTM5*, *CCR5*, *TDO2*, *FCER1G*, and *TLR8*. These findings suggest that a compressive analysis of eQTL and TME-based immune gene expression has a significant impact on cancer biology.

Through genome-wide association studies (GWASs), several germline variants associated with cancer susceptibility were identified through eQTL analysis. From the GTEx database, we found that the *PYGL* eGene was associated with the risk of cancer development. However, the minor allele frequencies of the previously identified variants, including rs1258966, rs72685323, and rs72685325, are relatively high in the East Asian population, ranging from 0.415 to 0.439. These eQTL might not be suitable predictive markers for East Asians.

Patients who have inherited defective genes in immune cells may experience different immunotherapy responses, clinical outcomes, and cancer risks [38]. These findings have implications for identifying individual eQTL based on germline variants and TME-based immune response genes. This study recognizes the association of 65 eQTL (60 germline eVariants in 22 TME-based immune response eGenes) and survival.

To understand the relationship between tumor features and cancer recurrence and tumor microenvironment-based immune response gene expression, we used RNA sequencing as a tool for analysis. We developed a 41-DEG panel to predict recurrence with an accuracy greater than 0.9. In this study, based on the current TME-based immune response gene expression, we offered a comprehensive analysis of immune response genes in the tumor invasion stage, tumor nodal stage, tumor sites, and tumor mutational burden. From the TME-based immune response gene expression data available for CRC, we have a clear biological interpretability and a basis for future clinical stratification and targeted interventions. The tumor nodal stage is suggestively correlated with *CD274* expression. Therefore, a high *CD274* expression may be a candidate for therapeutic strategies in stage III CRC. We also found that several genes, including *TYROBP*, *CD1C*, *KIR2DL3*, *CD3G*, and *TARP*, were significantly highly expressed in patients with a high tumor mutational burden. These genes may serve as onco-immunotherapy targets.

To identify the germline eVariants that affect the clinical outcome through TME-based immune response eGenes, we reviewed our results to find eQTL comprising survival-associated DEGs and eVariants. We found nine eQTL, including nine eVariants and two eGenes (*HGF* and *FCER1G*), that correlated with the survival of cancer patients. The *FCER1G* eGene is also associated with the tumor invasion stage, tumor nodal stage, and tumor site. *FCER1G*, located on chromosome 1, is essential for FcεRI signaling via an immunoreceptor tyrosine-based activation motif (ITAM). It activates the RAS/MAPK and NF-kB downstream pathways and increases intracellular calcium levels [39]. Antitumor activity has also been observed in *FCER1G* knockdown mice [40]. Immune response *FCER1G* expression in the TME is also associated with cancer prognosis [41]. We found that the eVariant rs2118867 could affect tumor features and clinical outcomes through the TME-based *FCER1G* eGene expression. However, in vitro cell migration and invasion assays need to be conducted. We further explored the chromatin effect predicted by DeepSEA in more detail. The results indicated that inherited rs12124509 slightly increased the binding preference of transcription factors such as *TAL1*, *GATA2*, and *TEAD4* (i.e., the eVariants generated new binding motifs for these chromatins) (Figure S6). From the eQTL analysis, we found that rs2118867 and rs12124509 germline eVariants are prognostic biomarkers in colorectal cancer patients. The *FCER1G* eGene is a potential therapeutic target. We identified novel cancer biomarkers for use in clinical practice.

The current study has some significant limitations. First, we used the TCGA and GTEx data to validate the higher expression of *FCER1G* in the tumor microenvironment (TME). At the protein level, we also demonstrated that higher *FCER1G* protein levels are associated with poor survival by the analysis of the Human Protein Atlas. However, we did not confirm the results in in vitro or in vivo studies, such as through Western blot RNA expression. Second, the RNA was isolated from primary CRC whole-slide cancer tissue from paraformaldehyde-fixed paraffin-embedded (FFPE). We did not use microdissection for tumor microenvironment (TME) enrichment. We used the Oncomine™ Immune Response Research Assay (OIRRA), designed to interrogate the tumor microenvironment (TME), for analysis. The OIRRA content included 395 genes and 36 across functional annotation groups associated with lymphocyte regulation, cytokine signaling, lymphocyte markers, checkpoint pathways, and tumor characterization. The assay was optimized to measure the expression of genes involved in tumor–immune interactions (https://www.thermofisher.com/order/catalog/product/A32881 (accessed on 5 January 2022)). We profiled bulk tumors to study the tumor microenvironment by functional annotation [42]. We did not use microdissection or spatial profiling against cross-contamination from cancer cells and the tumor environment (TME). In the future, we will perform single-cell RNAseq or spatial cell biology for TME tissue [43].

## 5. Conclusions

We conclude that a comprehensive analysis of eQTL in TME-based immune gene expression significantly impacts cancer biology and provides us with potential immune therapy targets.

## Figures and Tables

**Table 1:** Contributions of eVariants and eGenes to clinical outcome.

eGenes	Refseq	DGE*	eQTLs* (Number)	Gene Function	Hazard Ratio (95% CI)
*HGF*	NM_000601	0.0468	rs6962550/ rs11365736(2)	Cytokine signaling	5.49 [2.34–12.88]
*TLR8*	NM_138636	0.0029	rs12842223(1)	Lymphocyte infiltrate	4.23 [0.99–18.13]
*CCR5*	NM_001100168	0.0251	rs11456888(2)	Lymphocyte infiltrate	3.57 [1.39–9.14]
*LAPTM5*	NM_006762	0.0411	rs12121984/ rs694215/ rs12135225(8)	Lymphocyte infiltrate	3.51 [1.46–8.39]
*IL18*	NM_001562	0.0298	rs374315853(1)	T cell regulation	3.28 [1.11–9.7]
*TDO2*	NM_005651	0.0126	rs201071019(1)	Checkpoint pathway	2.92 [1.22–6.95]
*IFITM2*	NM_006435	0.0274	rs764825643(1)	Type I interferon signaling	2.9 [1.23–6.85]
*FCER1G*	NM_004106	0.0334	rs2118867/ rs12124509(7)	Lymphocyte infiltrate	2.85 [1.22–6.7]

Refseq: reference sequence; DGE*: differential gene expression; eQTLs*: eQTLs variants.

## Data Availability

The datasets used and analyzed during the current study are available from the corresponding author on reasonable request, and supplementary information files are available for this manuscript.

## Supplementary Materials

The following supporting information can be downloaded at: https://www.mdpi.com/article/10.3390/diagnostics12020315/s1. Figure S1: overlapping rate in GTEx and the expression level of frequently observed eGenes in different types of tissues. Figure S2: heatmap for cancer patients clustered by fuzzy K-means using 36 of the most prognostic immune response genes. Figure S3: performance of the DEGs and prognostic genes in the recurrence prediction model. Figure S4: predictive ability of the 36 most prognostic immune response genes and progression-free survival between the protective and risk groups. Figure S5: immune response genes associated with high FCER1G expression. Figure S6: impact of rs12124509 on chromatin features predicted by DeepSEA. Table S1: patient characteristics. Table S2: detail of all cis-eQTLs derived from matrix eQTL. Table S3: prognosis analysis, functional significance, and eQTL-probability of eVariants. Table S4: 41 differentially expressed genes. Table S5: prognostic significance genes. Table S6: nineteen eGenes that contribute to clinical outcome. Table S7: Multivariate cox regression analysis of survival.
