# Supplementary material for: The Expression Quantitative Trait Loci in Immune Response Genes Impact the Characteristics and Survival of Colorectal Cancer
Source: Diagnostics (Basel). 2022 Jan 26;12(2):315. doi: 10.3390/diagnostics12020315 (PMC8871427; doi:10.3390/diagnostics12020315)
Supplement: Supplementary file 1 [file diagnostics-12-00315-s001.zip › Additional file 1.pdf]

Figure S1

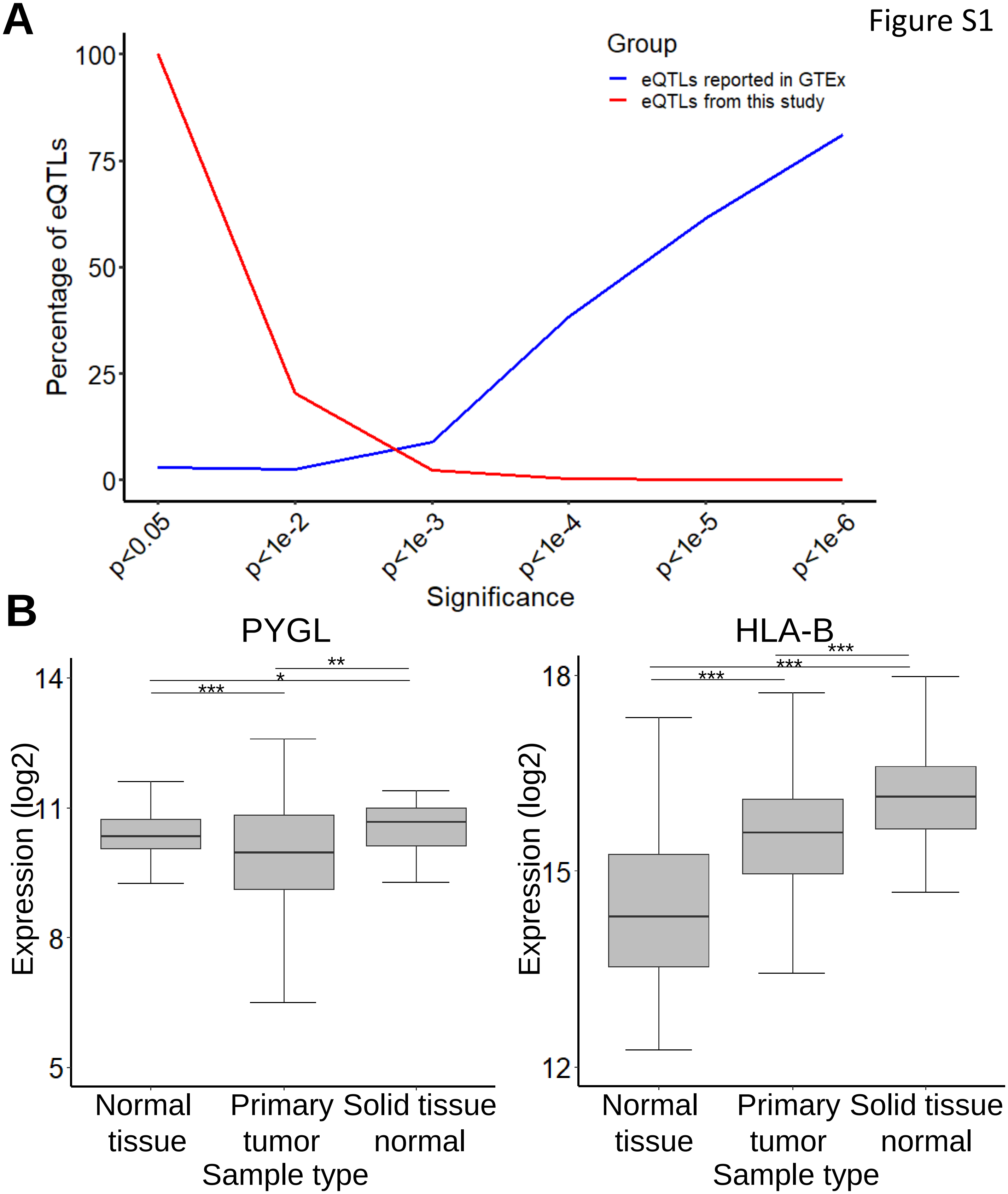

Figure S1. (**A**) Overlapping eQTL between this study and the GTEx database. The red line indicates the proportion of eQTL derived from our workflow under different levels of significance. The blue line indicates the proportion of overlapping eQTL found in the GTEx under different levels of significance. The higher the significance is, the higher the proportion of overlapping eQTL in the GTEx is. (**B**) Normalized expression of PYGL and HLA-B obtained from the Xena browser. The expression levels of both PYGL and HLA-B were higher in normal tissues adjacent to the tumor than in tumor tissues (solid, normal tissues). *$p<0.05$, **$p<0.01$, ***$p<1e-3$ from the Wilcoxon rank-sum test.

Figure S2

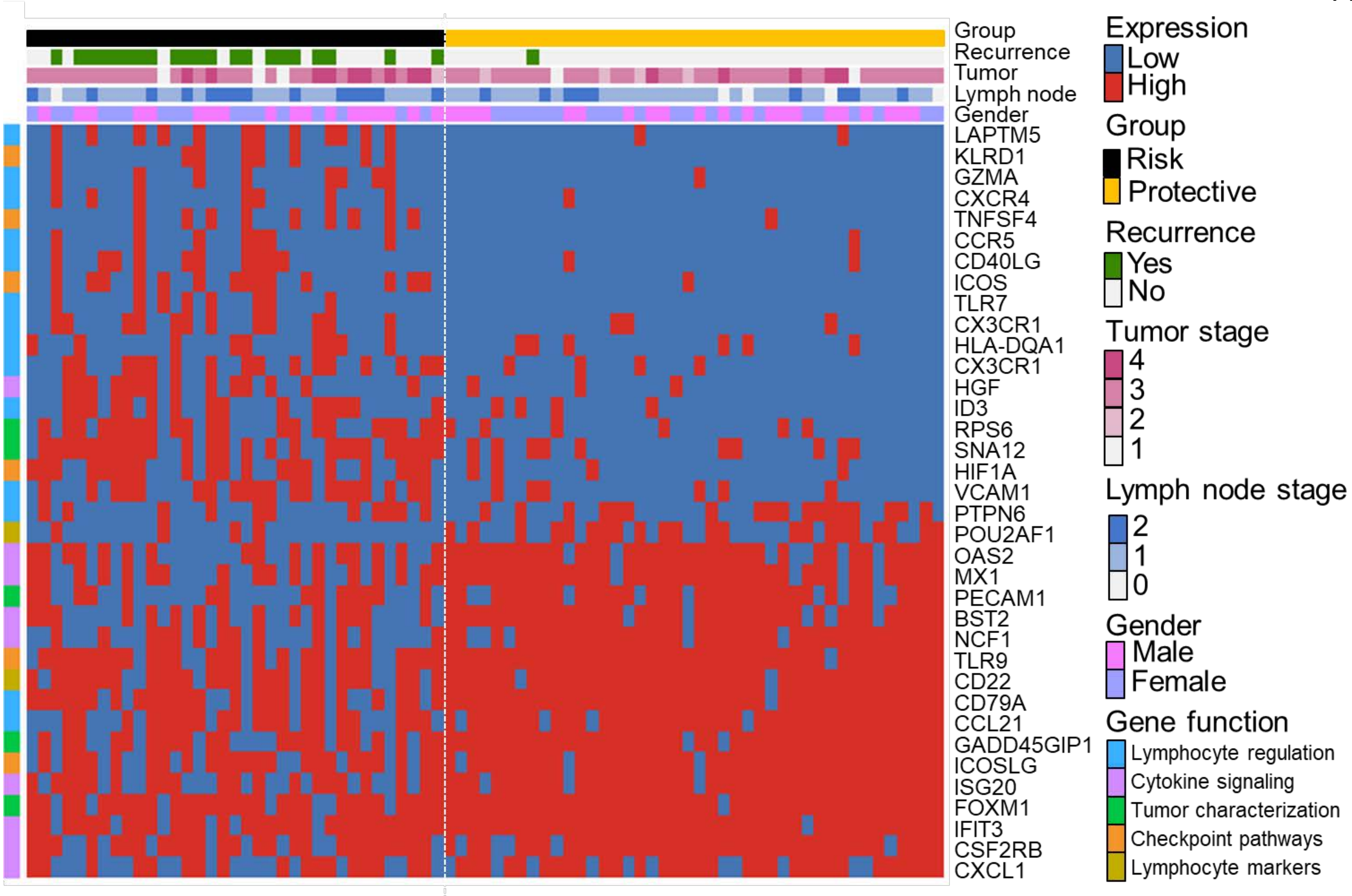

Figure S2. Heatmap for cancer patients clustered by fuzzy K-means using 36 of the most prognostic immune response genes. In comparison with the protective group (orange), some lymphocyte regulatory factors were upregulated while some cytokine signaling factors were downregulated in the risk group (black).

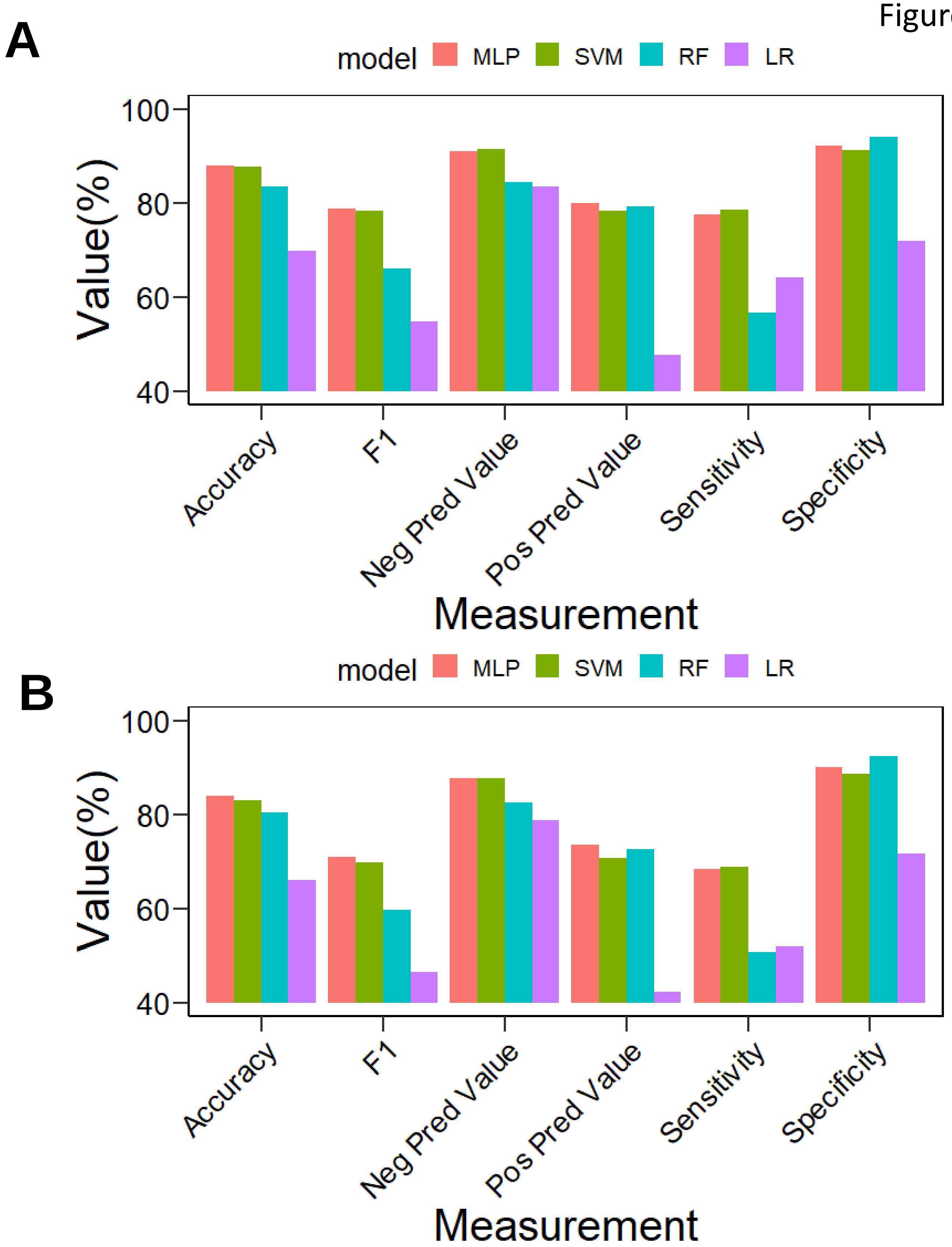

Figure S3. Performance of the DEGs and prognostic genes in the recurrence prediction model. Measurement of different models built with (**A**) 41 dichotomized DEGs and (**B**) 36 of the most prognostic immune response genes. The results were evaluated in terms of the accuracy, F1-score, negative predictive value, positive predictive value, sensitivity and specificity.

Figure S4

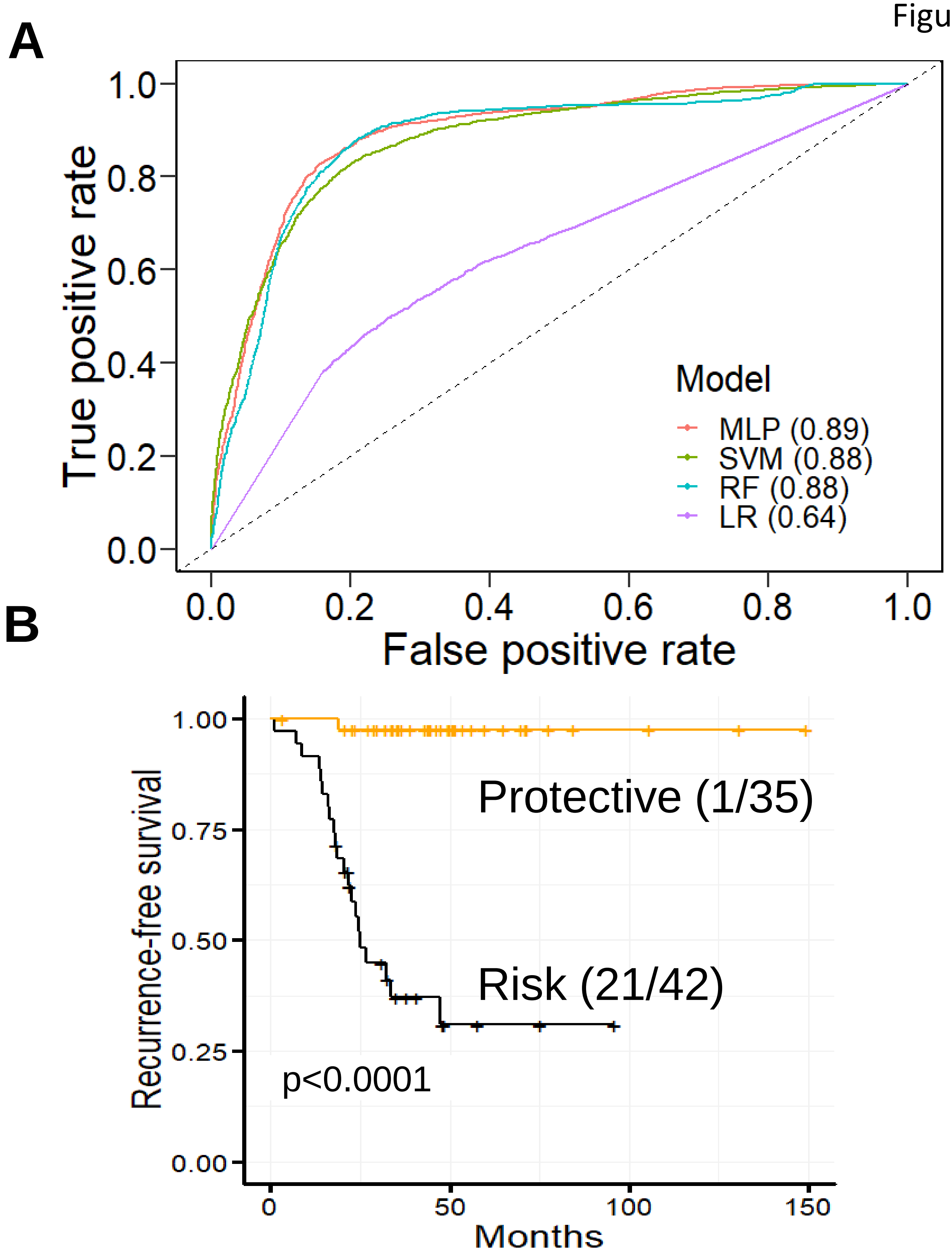

Figure S4. Predictive ability of the 36 most prognostic immune response genes and progression-free survival between the protective and risk groups. (**A**) Receiver operating characteristic (ROC) curves and relative areas under the ROC curves (AUROCs) of different classification models. Classification models were built from the 36 most prognostic genes. Most of these strategies performed well, with an AUROC of approximately 0.88, except for the LR. (MLP: multilayer perceptron; SVM: support vector machine; RF: random forest; LR: logistic regression) (**B**) Kaplan-Meier curves between the protective and risk groups. The protective group with a good prognosis is marked as the orange line, while the risk group with a poor prognosis is marked as the black line.

Figure S5

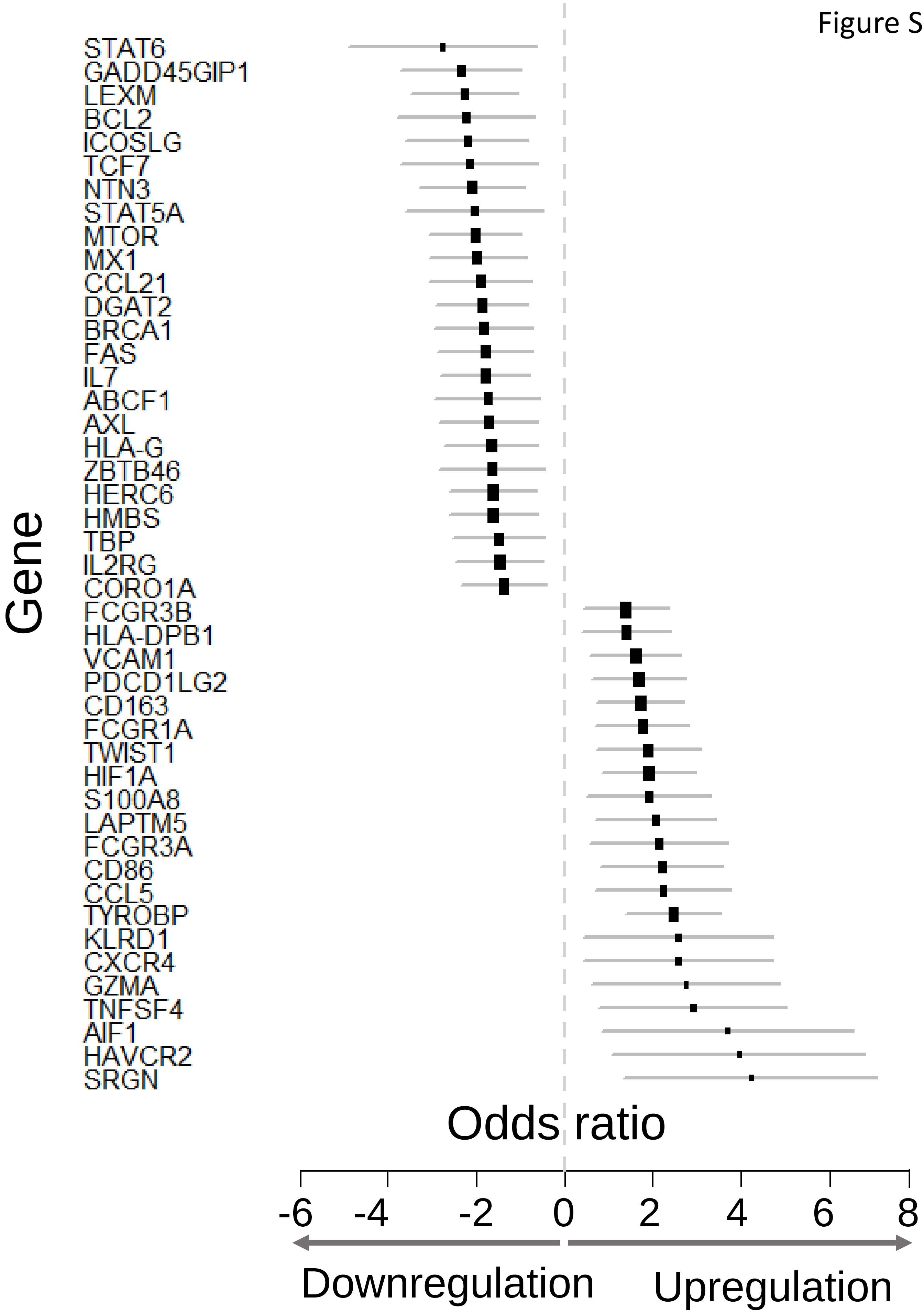

Figure S5. Forest plot of the odds ratios for immune response gene expression in samples with high FCER1G expression.

Figure S6

A

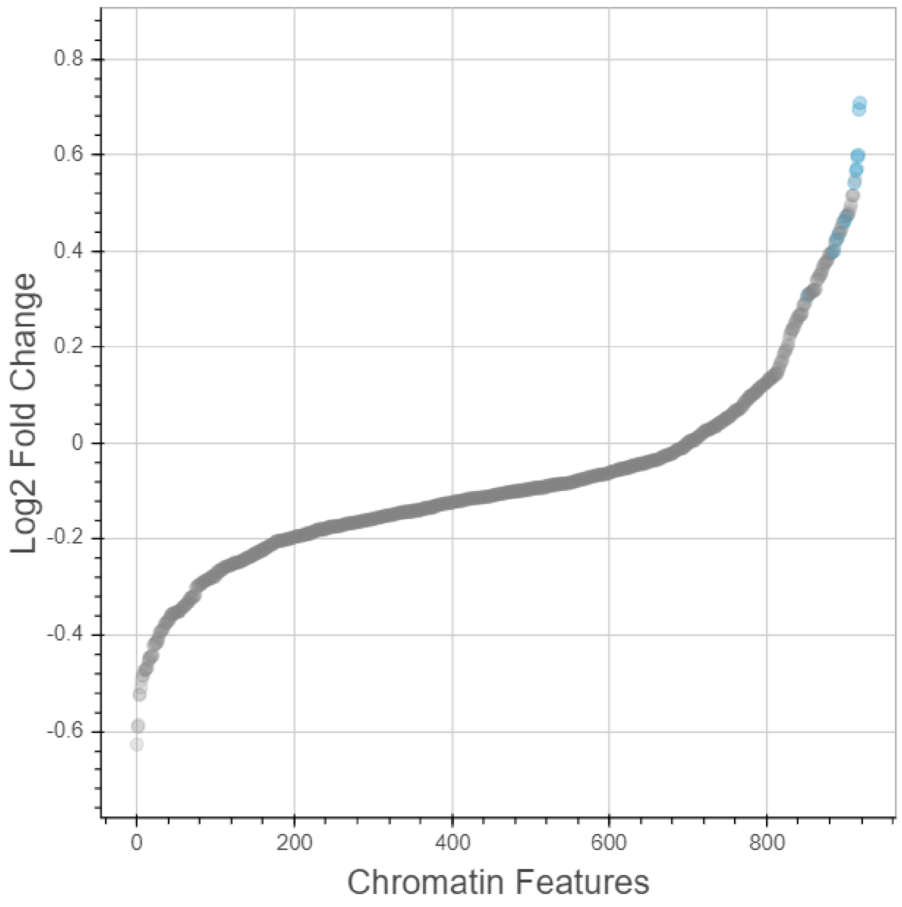

B

Functional significance score: 5.9093e-2

Chromat...	Cell type	Treat...	*Effect (L...*	E-value	P(Reference)	P(Alternative)
**TAL1**	**K562**	**None**	**0.70808**	**0.007919**	**0.028884**	**0.048816**
**GATA2**	**K562**	**None**	**0.69444**	**0.009732**	**0.018076**	**0.030803**
**TEAD4**	**K562**	**None**	**0.60041**	**0.008964**	**0.040814**	**0.062976**
**p300**	**K562**	**None**	**0.59592**	**0.008937**	**0.038864**	**0.059565**
**STAT5A**	**K562**	**None**	**0.57141**	**0.007740**	**0.013421**	**0.020596**
**TBLR1**	**K562**	**None**	**0.56662**	**0.009888**	**0.010420**	**0.015995**
GATA1	PBDEFetal	None	0.54856	0.013235	0.001717	0.002605
**ARID3A**	**K562**	**None**	**0.54173**	**0.009053**	**0.011270**	**0.016824**
CCNT2	K562	None	0.51637	0.013700	0.007265	0.010728
eGFP-GA...	K562	None	0.51561	0.015224	0.010320	0.015250
GATA1	K562	None	0.51551	0.025050	0.001215	0.001824
GATA1	PBDE	None	0.49776	0.010236	0.028722	0.041166
GATA2	K562	None	0.49229	0.026818	0.003489	0.005163
NR2F2	K562	None	0.48423	0.013354	0.014897	0.021436
SIRT6	K562	None	0.47776	0.014450	0.002750	0.003981
ATF1	K562	None	0.4772	0.023078	0.009683	0.013908
**Brg1**	**K562**	**None**	**0.47294**	**0.003142**	**0.009867**	**0.013920**
c-Myc	K562	IFNg30	0.47218	0.023373	0.010805	0.015411
c-Jun	K562	IFNa6h	0.47186	0.028153	0.001339	0.001910
**PML**	**K562**	**None**	**0.46283**	**0.008684**	**0.018240**	**0.025861**

Figure S6. Impact of chromatin features predicted by DeepSEA.

(**A**) Distribution of chromatin features and fold change in binding preference. The blue point indicates a significant effect (E-value<0.01).

(**B**) The table shows detailed information from DeepSEA.
