# Supplementary material for: The Expression Quantitative Trait Loci in Immune Response Genes Impact the Characteristics and Survival of Colorectal Cancer
Source: Diagnostics (Basel). 2022 Jan 26;12(2):315. doi: 10.3390/diagnostics12020315 (PMC8871427; doi:10.3390/diagnostics12020315)
Supplement: Supplementary file 1 [file diagnostics-12-00315-s001.zip › Additional file 2.pdf]

Table S1. Patient characteristics

Characteristic	Recurrence (N=22)	Non-recurrence (N=55)	P value
Age			0.269
<65	13(59.1%)	41(74.5%)	
>=65	9(40.9%)	14(25.5%)	
Gender			0.99
Male	11(50%)	28(50.9%)	
Female	11(50%)	27(49.1%)	
Tumor location			0.765
Left	18(81.8%)	41(74.5%)	
Right	4(18.2%)	12(21.8%)	
Tumor invasion stage			0.715
T1/T2	2(9.1%)	8(14.5%)	
T3/T4	20(90.9%)	47(85.5%)	
Tumor nodal stage			0.582
N0/N1	14(63.6%)	40(72.7%)	
N2	8(36.4%)	15(27.3%)	
Tumor mutation Burden			0.99
Hypermutation	2(9.1%)	6(10.9%)	
Non-hypermutation	20(90.9%)	49(89.1%)	
KRAS mutation			0.99
Mutation	8(36.4%)	20(36.4%)	
Wild type	14(63.6%)	35(63.6%)	
TP53 mutation			0.99
Mutation	20(90.9%)	50(90.9%)	
Wild type	2(9.1%)	5(9.1%)	
